# Comparison of Various Chromatographic Systems for Analysis of Cytisine in Human Serum, Saliva and Pharmaceutical Formulation by HPLC with Diode Array, Fluorescence or Mass Spectrometry Detection

**DOI:** 10.3390/molecules24142580

**Published:** 2019-07-16

**Authors:** Karol Wróblewski, Anna Petruczynik, Tomasz Tuzimski, Dominika Przygodzka, Grzegorz Buszewicz, Patrycjusz Kołodziejczyk, Piotr Tutka

**Affiliations:** 1 Department of Experimental and Clinical Pharmacology, University of Rzeszów, Kopisto 2a, 35-959 Rzeszów, Poland; 2 Department of Inorganic Chemistry, Medical University of Lublin, Chodźki 4a, 20-093 Lublin, Poland; 3 Laboratory for Innovative Research in Pharmacology, University of Rzeszów, Kopisto 2a, 35-959 Rzeszów, Poland; 4 Department of Physical Chemistry, Medical University of Lublin, Chodźki 4a, 20-093 Lublin, Poland; 5 Department of Forensic Medicine, Medical University of Lublin, 8b Jaczewskiego St, 20-090 Lublin, Poland; 6 National Drug and Alcohol Research Centre, University of New South Wales, Sydney, NSW 2031, Australia

**Keywords:** cytisine, HPLC-DAD, HPLC-FLD, HPLC-MS/MS, SPE, optimisation of chromatographic systems, serum, saliva, pharmaceutical preparation, retention mechanism

## Abstract

*Background:* Identification and quantitative determination of cytisine, especially in biological samples and pharmaceutical formulations, is still a difficult analytical task. Cytisine is an alkaloid with a small and very polar molecule. For this reason, it is very weakly retained on reversed phase (RP) stationary phases, such as commonly used alkyl-bonded phases. The very weak retention of cytisine causes it to be eluted together with the components of biological matrices. *Objective:* Comparison and evaluation of various chromatographic systems for analysis of cytisine in different matrices—serum, saliva and pharmaceutical formulation—by high performance liquid chromatography (HPLC) with diode array (DAD), fluorescence (FLD) and mass spectrometry (MS) detection. *Methods:* The analyses were performed using HPLC in reversed phase (RP), hydrophilic interaction liquid chromatography (HILIC) and ion exchange chromatography (IEC) modes. Different sample pre-treatment methods were tested: Protein precipitation (with acetone, methanol (MeOH) or acetonitrile (ACN), and solid phase extraction (SPE) using cartridges with octadecyl (C18), hydrophilic-lipophilic balanced copolymer (HLB) or strong cation exchange sorbents (Strata X-C). *Conclusion:* Significant differences were observed in retention parameters with a change of the used chromatographic system. The various properties of stationary phases resulted in differences in analyte retention, peaks’ shape and systems’ efficiency. The weakest retention was observed using RP systems; however, the use of the Polar RP phase can be an alternative for application in green chromatography. In the strongest retention was observed using a strong cation exchange (SCX) phase. The most optimal systems were chosen for the analysis of cytisine in the pharmaceutical preparation, serum and saliva after sample pre-treatment with the new SPE procedure. Due to the sensitivity, the use of HPLC-DAD or HPLC-FLD is the most optimal for drug analysis in pharmaceutical preparations, whereas HPLC-MS is suitable for analysis of cytisine in biological samples.

## 1. Introduction

Cytisine is a quinolizidine alkaloid originating from plants of the *Leguminosae* (*Fabaceae*) family. The greatest amount of the alkaloid was found in the seeds of *Laburnum anagyroides* (Golden Rain acacia; about 1–5%) [1]. Plants containing cytisine have been used as a natural remedy and a medicinal agent for various purposes for centuries [2,3].

Cytisine is a nicotinic acetylcholine receptors (nAChRs) partial agonist with a high affinity to the brain α4β2 and α6β2 nAChRs subtype [3,4]. The alkaloid has been widely used for nicotine addiction treatment in Central and Eastern Europe, Central Asia and Canada [5]. Cytisine is marketed in a tablet form as Tabex^®^ (in many countries in Europe and Central Asia) and Cravv^®^ (in Canada) or capsule form as Desmoxan^®^ (in Poland).

Clinical trials have shown that cytisine is more effective than placebo and nicotine replacement therapy (NRT) for smoking cessation [6,7,8]. The advantage of cytisine therapy is a much lower cost compared to other drugs used for smoking cessation. Therefore, cytisine is currently considered an inexpensive, effective and safe alternative for the currently available antismoking treatment of nicotine addiction, and is considered a first-line pharmacotherapy for smoking cessation in countries where access to combined NRT or varenicline is limited due to availability or cost [3,9,10].

The standard dosing of cytisine has been 1.5 mg orally taken every 2 h for up to six doses (9 mg) per day for three days, with progressive tapering to two tablets per day over 25 days. The dosing regimen is complex; however, cytisine has the shortest treatment duration of any of the currently approved smoking cessation medications (e.g., 12 weeks for varenicline: or eight weeks for NRT) [7,11,12]. To date, several methods have been developed for the determination of cytisine in plant material [13,14,15,16], rat brain [17,18], serum (limit of detection (LOD) was 4.0 ng mL^−1^, 12 ng mL^−1^) [19,20], plasma (limits of quantification (LOQs) were 1 ng mL^−1^, 0.522 ng mL^−1^ or 2.97 pg on column, respectively) [17,18,21,22,23], whole blood [24], urine (LOD was 50 ng mL^−1^, LOQs were 7.1 ng mL^−1^, 0.108 μg mL^−1^) [20,22,23,25], biological material collected posthumously (including blood, bile, stomach contents, liver, brain) [26] and pharmaceutical formulations [23]. Some of the developed methods have been prepared for simultaneous determination of multiple alkaloids [14,15,16,20,21,24], and one method has been applied only to their detection [25]. Most of the described methods relate to the detection and/or quantification of cytisine in poisoning with plant material or overdose in humans [20,21,24,25,26,27].

**Figure 1:**
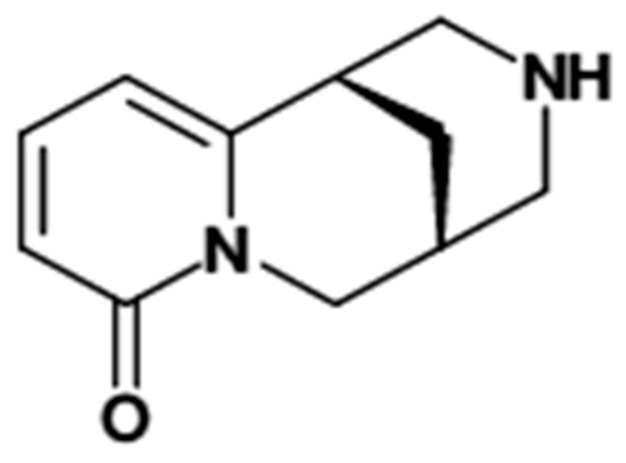
Cytisine structure.

Cytisine (Figure 1) is a small, polar, basic compound (pKa = 7.92) whose physicochemical properties are still studied [28,29]. Cytisine has long history of use; however, knowledge on its chromatographic properties is still incomplete. Based on the literature data, it was found that analysis of cytisine in biological samples and pharmaceutical formulations was carried out using reversed phase chromatography, mainly octadecyl (C18) stationary phase and water-organic mobile eluents with acidic pH. In these systems cytisine is very weakly retained.

Recently, the pharmacokinetics of cytisine in humans have been described [23,30,31]. In these studies, cytisine was measured in plasma and urine after a single oral dose of 3 mg [23], after repeated administrations during the standard dosing regimen [30] and after a single oral dose of 1.5, 3 and 4.5 mg [31]. The quantitative determination of cytisine in human and animal biological samples has been carried out by high-performance liquid chromatography with UV detection (HPLC-UV) [19] or by HPLC coupled with mass spectrometry (HPLC-MS) or tandem mass spectrometry (HPLC-MS/MS) [20,21,22,23,24,25,26,30,31]. The HPLC method with UV detection was used for determination of cytisine in pharmaceutical formulation [23]. Analysis of cytisine in human biological samples and formulations were performed by reverse phase chromatography using octadecyl (C18) [20,22,23,24,26,30,31] or octyl (C8) [21,25] stationary phases with aqueous eluents contained acetonitrile (MeCN) as organic modifier and ammonium formate buffer, ammonium acetate buffer, formic acid or phosphate buffer. For sample pre-treatment prior to chromatographic analysis, liquid–liquid extraction (LLE) [19,22,25,26], solid phase extraction (SPE) [20,23,30,31] or only the protein precipitation was used [23]. In the SPE technique, C18 [20,23], HLB [24] and mixed mode SPE [21] cartridges were used. Often, the recoveries in the proposed extraction procedures were not too high. For example, in the method of simultaneous detection of 22 alkaloids in human urine, cytisine recovery was only 15% [25].

There is one published description of the method used for quantification of cytisine in clinical samples (human plasma) following administration of pharmaceutical formulations contained the drug [23]. HPLC method with MS detection using electrospray ionization (ESI) was applied. Cytisine was analysed using a Phenomenex C18 (4.6 × 150 mm, 5 μm) column and eluted with a mobile phase containing acetonitrile and ammonium formate buffer at pH 4.5. Sample pre-treatment was performed by protein precipitation method with methanol (MeOH). The absolute cytisine recovery obtained by the method was, on average, 75%. The method was applied for pharmacokinetics study of cytisine, in healthy smokers following a single dose of 3 mg [23] and also for plasma concentrations of cytisine in healthy adult smokers taking recommended doses [30]. This method, after appropriate modification, was applied for quantification of cytisine in plasma after single oral dose of 1.5, 3 or 4.5 mg. A stationary phase Gemini C18 (4 × 100 mm, 5 μm) column was used [31].

Accordingly, there is need for further study on the retention mechanism of cytisine in the different chromatographic conditions.

The aim of this study is to compare and evaluate various chromatographic systems for analysis of cytisine in different matrices—serum, saliva and pharmaceutical formulation—by HPLC with diode array (DAD), fluorescence (FLD) and mass spectrometry detection (MS). For the first time, detection of cytisine in human saliva is also performed.

## 2. Results and Discussion

### 2.1. Selection of Condition of Chromatographic Systems and Detection Techniques

Cytisine standard was chromatographed using different stationary phases (Table 1) in various eluent systems to choose the optimal chromatographic conditions for analysis of the investigated compound in biological samples and pharmaceutical preparations. For this purpose, retention, separation selectivity, peaks’ shape and systems’ efficiency obtained in various chromatographic systems were compared.

#### 2.1.1. Selection of Detection Techniques

**Figure 2:**
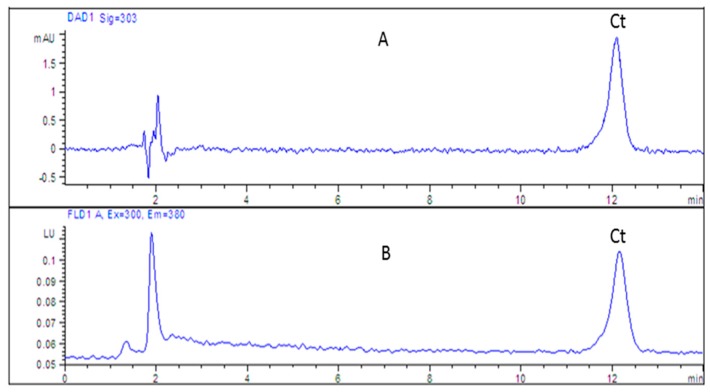
Chromatograms obtained for cytisine (Ct) standard (1 µg/mL) using HPLC-DAD (**A**) and HPLC-FLD (**B**) techniques. Stationary phase: Luna 5 μm, SCX 100A, 150 × 4.6 mm column; mobile phase: 15% ACN and 100 mM of formate buffer at pH 4.0.

Different detection techniques were tested including diode array (DAD), fluorescence (FLD) and mass spectrometry (MS). The fluorescence detector is one of the most sensitive detectors in liquid chromatography. Fluorescent properties of cytisine were studied [29]; however, no fluorescence detection has been used so far. The different excitations (λexc) and emission wavelengths (λem) were tested for cytisine analysis in our study. λexc = 300 nm and λem = 380 nm (the maximum of the emission band) was selected as the most optimal parameters for cytisine monitoring. However, the intensity of the signal was comparable to that obtained with the DAD detection at the maximum wavelength at about 303 nm (Figure 2).

LC-MS/MS is generally preferred for the analysis of compounds in various samples, especially in biological samples. Chromatographic systems with a mobile phase containing volatile constituents are compatible with MS. The most optimal systems according to chromatographic parameters and mobile phases containing volatile constituents were selected for analysis of cytisine by LC-MS/MS.

#### 2.1.2. Comparison of Chromatographic Systems

Cytisine, as a very polar basic compound, can strongly interact with free silanol groups present on the surface of chemically-bonded stationary phases. It leads to poor separation efficiency, asymmetric peaks and difficulties in reproducibility of analysis. To avoid these disadvantageous interactions the following methods are used: suppression of analyte ionization, suppression of silanol ionization by the use acid or buffer at appropriate pH, the use of ion-pair reagents as eluent additives, silanol blockers and the use of other phases than octadecyl stationary phases. Good alternative for analysis of polar basic compounds in RP system is ion-exchange chromatography. We examined chromatographic systems suitable for analysis of basic compounds; experiments were performed especially on phenyl stationary phases suitable to π–π interactions with mobile phases at acidic pH when ionization of free silanol groups are suppressed or with addition of silanol blockers (diethylamine or ionic liquids). Ion-exchange chromatography was also successfully applied for analysis of cytisine. In our experiments various compositions of mobile phases were applied depending on properties of used stationary phases.

##### Analysis of Cytisine on Octadecyl Stationary Phase

The experiments started with the investigation of chromatographic parameters obtained for cytisine standard solution (10 µg mL^−1^), using the Hydro RP column with the most often applied alkyl-bonded stationary phase. Cytisine was very weakly retained using this stationary phase. For this reason, it was necessary to apply mobile phases containing only 5% organic modifier in water or only water with appropriate additives for reduction of peak tailing: formic acid, diethylamine (DEA), sodium salts or ionic liquids (ILs). In these systems cytisine was still weakly retained and obtained peaks were very asymmetrical (Table 2). In most often applied systems, the alkaloid was eluted after less than 2 min. The strongest retention of cytisine was obtained in the system with the mobile phase containing water and 0.025 ML^−1^ of 1-butyl-3-methylimidazolium tetrafluoroborate (retention time t_R_ = 3.46), but the peak was still asymmetrical. The octadecyl stationary phase proved to be the least useful for analysis of cytisine, with all the tested phases; very weak retention, asymmetrical peaks and low N/m values were obtained.

##### Analysis of Cytisine on Phenyl Stationary Phases

Because a very weak retention, asymmetrical peaks and poor system efficiency were obtained on the Hydro RP column with the octadecyl stationary phase, in the next step of investigations Phenyl-Hexyl and Polar RP columns with phenyl moieties were applied. The introduction of π–π active aromatic moieties to the n-alkyl chain RP sites generates a concerted π–π retention mechanism, which, as a consequence of the new functionality, diversifies the common RP interaction properties without altering the latter severely. The π–π interactions typically involve the charge-transfer of electrons from electron-rich (π-base) to electron-poor (π-acid) substances. The π–π interactions can also involve a simple overlap of π-orbitals in two interacting molecules. The introduction of additional interactions may result in an increase in compound retention and, due to preferential π–π interactions reducing the deleterious effect of free silanols, may lead to improved peak shape and system efficiency [32,33].

**Figure 3:**
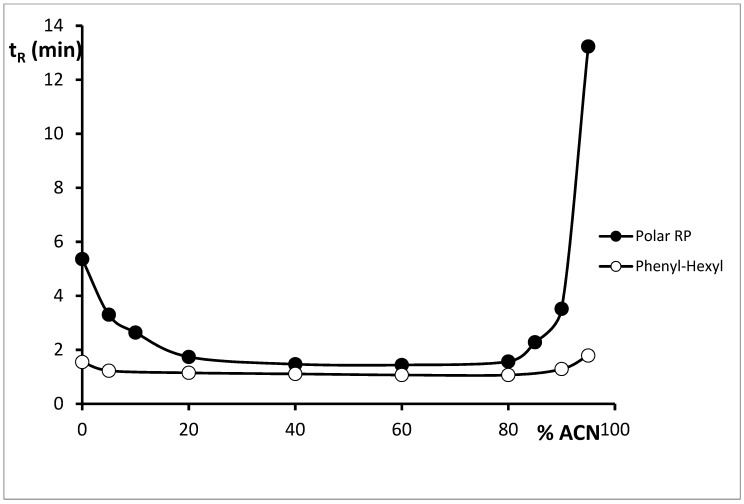
Dependence of t_R_ vs. ACN concentration obtained using Synergi HST Polar RP and Charged Surface Hybrid (CSH) Phenyl-Hexyl columns with mobile phase containing ACN, H_2_O + 0.1 % HCOOH.

The similar mobile phase compositions, as those used on the alkyl-bonded stationary phase, were applied on columns with phenyl moieties. Application of the Phenyl-Hexyl column with mobile phases containing HCOOH, 5% MeOH or 5% MeCN, or without organic modifier, resulted also in very weak retention of cytisine and asymmetrical peaks. The relationship between cytisine retention and concentration of acetonitrile in aqueous mobile phase containing addition of 0.1% formic acid was also investigated. The retention of cytisine initially decreased with the increase of acetonitrile concentration, then the retention increased with the increase of acetonitrile concentration. The dependence between t_R_ and acetonitrile concentration was not linear using the Phenyl-Hexyl column, but showed a U-shape relationship typically for ionic analytes on stationary phases with π–π interactions (Figure 3). At lower percentages of organic modifier, solute retention resembled that of classical reversed-phase systems. At higher percentages of organic modifier; however, a more typical behaviour of normal-phase separations was observed with increasing proportion of organic modifier, but the mechanism for the “U-shape” retention was still investigated. However, in all concentration ranges of acetonitrile, cytisine was weakly retained and peaks were asymmetrical. The application of mobile phases containing 5% MeOH or 5% MeCN, or without organic modifier, acetate buffer at pH 3.5 and 0.025 ML^−1^ DEA resulted in the increase of cytisine retention and a slight improvement in the shape of peaks. However, peaks were still asymmetrical. The asymmetry factor values (As) ranged between 0.60 and 0.73. The application of the mobile phase containing 0.025 ML^−1^ of NaCl resulted in a decrease of retention and deterioration of peak shape, while in the mobile phase with sodium tetrafluoroborate, retention significantly increased but the symmetry of the peak was still unacceptable. The symmetrical peaks on the column were only obtained when mobile phases with addition of 1-butyl-3-methylimidazolium chloride or 1-butyl-3-methylimidazolium tetrafluoroborate were applied (as was 1.04 and 1.08, respectively). Retention time for cytisine was 2.45 min in the system with 1-butyl-3-methylimidazolium chloride and 3.25 min in the system with 1-butyl-3-methylimidazolium tetrafluoroborate.

**Figure 4:**
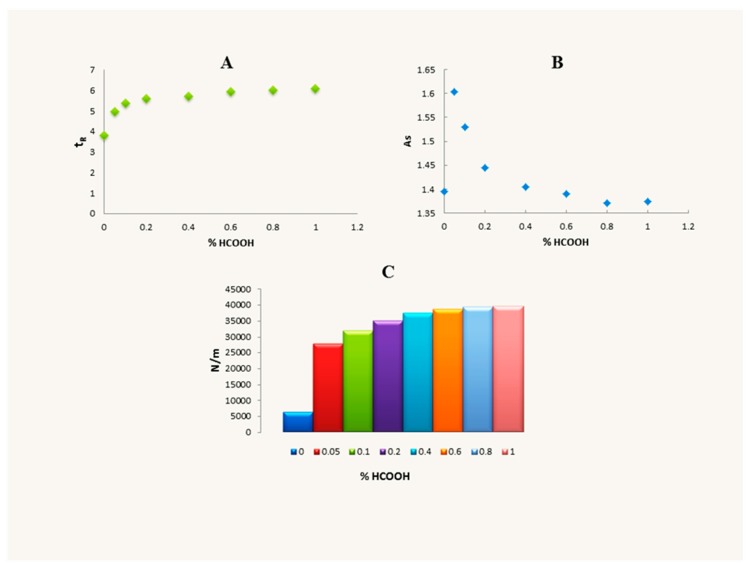
Relationship between formic acid concentration and retention time (t_R_) (**A**), asymmetry factor (As) (**B**) and system efficiency (N/m) (**C**). Data was obtained for cytisine standard (10 µg/mL) using Synergi 4 µm Polar-RP 80 Å column with mobile phase containing H_2_O and HCOOH.

An increase of cytisine retention and improvement of peaks’ shape in almost all applied mobile phases were observed on the Polar RP column. For this reason, on the column more mobile phases were tested in order to choose the optimal system for analysis of cytisine. In systems with 5% of MeCN or MeOH and addition of HCOOH, retention was higher compared to the retention obtained on two previously used columns, but peaks were still asymmetrical. U-shape dependence between cytisine retention and acetonitrile concentration was also obtained using this column. The application of the mobile phase containing only water and formic acid allowed for the improvement of peak symmetry. The dependence between formic acid concentration and t_R_, As and the number of theoretical plates per meter (N/m) was also examined (Figure 4). The retention of cytisine increased with the increase of formic acid concentration from 0% to 0.1%. Further increase of formic acid concentration caused only a slight increase of cytisine retention. The increase in formic acid concentration resulted in the improved shape of the peaks and systems’ efficiency. The improvement of results was observed especially in the range from 0.05% to 0.6% of formic acid. Such a phenomenon can be explained by the ionic interaction of the protonated analyte with oppositely charged species, which results in either the formation of stable ion pairs or the disruption of the analyte solvation. In pure water, applied as a mobile phase, the peak obtained for cytisine was symmetrical but the system efficiency was poor. In mobile phases both with organic modifier and without modifier and addition of DEA, cytisine peaks were symmetrical and t_R_ were in the range from 3.49 to 5.90 min. N/m values in systems with DEA were higher than 20,000 in all cases.

In the next step of experiments mobile phases containing sodium salts were tested as mobile phase additives for receiving the highest retention, more symmetrical peaks and improvement of systems’ efficiency. The retention of cytisine increased with application of salts with anions systematised according to Hofmeister’ series. The values of t_R_ increased from 3.76 min in the system with sodium dihydrogen phosphate to 8.95 min in the system with sodium hexafluorophosphate. In systems with addition of sodium salts, further significant improvement of peaks’ symmetry and systems’ efficiency were also observed. In all mobile phases with various sodium salts peaks were symmetrical. Asymmetry factor value obtained in the system with sodium dihydrogen phosphate was 1.22. Symmetry of peaks improved systematically according to the location of salt anions in Hofmeister series. In the system containing sodium hexafluorophosphate, As value was 1.04. Similar dependence was observed for N/m values obtained for cytisine. The lowest N/m value was obtained when the mobile phase containing sodium dihydrogen phosphate was applied (41,870), the highest in the system with sodium hexafluorophosphate (69,470). To choose the optimal chromatographic systems for analysis of cytisine, mobile phases containing ILs with 1-butyl-3-methylimidazolium cation and the same anions as in sodium salts were examined. In systems with ILs, weaker retention, worse shape of peaks and lower N/m values were obtained in almost all cases compared to systems with sodium salts containing the same anions. For example, in chromatographic systems with sodium dihydrogen phosphate, t_R_ = 3.76, As = 1.22 and N/m = 41870 were obtained, while in system with 1-butyl-3-methylimidazolium dihydrogen phosphate, t_R_ = 2.66, As = 1.97 and N/m = 16610 were determined. Only in the system with ILs composed of anions from the end of Hofmeister series (chlorate and hexafluorophosphate anions) were very symmetrical peaks and good systems’ efficiency achieved. Taking into account all mobile phases tested on the Polar RP column in terms of retention, peak shape and systems’ efficiency, the best results were obtained in systems with addition of sodium salts, especially containing anions from the end of Hofmeister series (NaBF_4_, NaClO_4_ and NaPF_6_). Good results were obtained in systems with ILs composed with chlorate and hexafluorophosphate anions, and also in systems with the addition of DEA.

Application of chromatographic systems with double protection against undesirable interactions of basic analytes with free silanol groups: phenyl stationary phase with π–π interaction and mobile phase with addition of acid, acidic buffer and especially DEA, or ILs as free silanol blockers, leads to the obtainment of high system efficiency, symmetrical peaks and the strongest retention compared to the octadecyl stationary phase.

##### Analysis of Cytisine by HILIC

For further optimisation of the chromatographic system, for the analysis of cytisine, hydrophilic interaction liquid chromatography (HILIC) was applied. HILIC provides an alternative approach to effectively separation of small polar analytes on polar stationary phases [34]. In HILIC, polar stationary phases are applied with aqueous mobile phases containing a high concentration of organic modifier. The mechanism of HILIC separation is complicated and involves various combinations of hydrophilic interactions, ion exchange and reversed-phase retention. HILIC is suitable for the analysis of polar compounds including polar-neutral and polar-ionised analytes. In our experiments, three columns with different properties were applied for cytisine analysis: ACE HILIC-A with silica stationary phase, ACE HILIC-B with aminopropyl and ACE HILIC-N with polyhydroxy stationary phase. Various concentrations of acetonitrile in mobile phases containing addition of formic acid, ammonium formate or formate buffer at pH 4.0 were tested. Mobile phase containing 80% of the organic modifier was optimal in terms of retention, peaks’ shape and systems’ efficiency for analysis of cytisine on all HILIC columns. The dependence on t_R_, As and N/m values of acetonitrile concentration in aqueous mobile phases containing 0.1% of formic acid was studied. A typical, for the HILIC mode, increase in cytisine retention with the increase of acetonitrile concentration was obtained. Peaks symmetry deteriorated with increasing acetonitrile concentration, while N/m values increased in higher concentrations of acetonitrile. Application of HILIC-A and HILIC-B columns led to obtaining significantly stronger retention of cytisine in the system with formate buffer compared to the system with ammonium formate. The peak obtained for cytisine was very asymmetrical using the HILIC-A column with the mobile phase containing ammonium formate, whereas the system with buffer was significantly more optimal. As in the system was 1.08 and system efficiency was also higher (N/m = 56940). Great differences in retention time were observed for both tested mobile phases using the HILIC-B column with the aminopropyl phase. Cytisine was weakly retained (t_R_ = 2.96 min) in the system with ammonium formate, whereas in the system with formate buffer t_R_ increased to 9.61 min. As values were 0.80 and 1.51, respectively. Low N/m values were obtained on the column in both systems, especially in the mobile phase with buffer (only 4680 N/m). The chromatographic parameters obtained using the HILIC-N column were similar in both mobile phases; however a slightly better result was obtained when formate buffer was added to the eluent. Based on the experiments in HILIC mode, it can be concluded that the most optimal chromatographic parameters for analysis of cytisine were obtained on the HILIC-A column with the mobile phase containing formate buffer. Systems with both tested mobile phases applied on the HILIC-N column can also be useful for determination of the alkaloid.

HILIC systems can also be useful for determination of the small polar molecule of cytisine, the best result was especially obtained on the HILIC-A column with the mobile phase containing 80% ACN and formate buffer at pH 4.0.

##### Analysis of Cytisine by Ion-Exchange Chromatography

Another chromatographic method for the analysis of polar ionisable compounds is ion exchange chromatography. For analysis of cytisine, the SCX column and mobile phases containing phosphate buffer at pH 2.5 or formate buffer at pH 4.0 were examined. Cytisine was strongly retained on the column with mobile phases containing only buffer. For this reason, it was necessary to use mobile phases containing the organic modifier. The more symmetrical peaks were obtained in systems with acetonitrile compared to systems with methanol and; thus, in the next steps of experiments, mobile phases with acetonitrile were applied. Cytisine was significantly strongly retained when the mobile phase with 15% of acetonitrile contained formate buffer compared to the system with phosphoric buffer (t_R_ equal 48.62 and 5.13 min, respectively). In both chromatographic systems, excellent symmetry of peaks were obtained (As = 0.96 and 1.04, respectively). The high N/m values were also obtained in both mobile phases; however, the higher value was observed in the system with formate buffer (N/m > 75,000).

**Figure 5:**
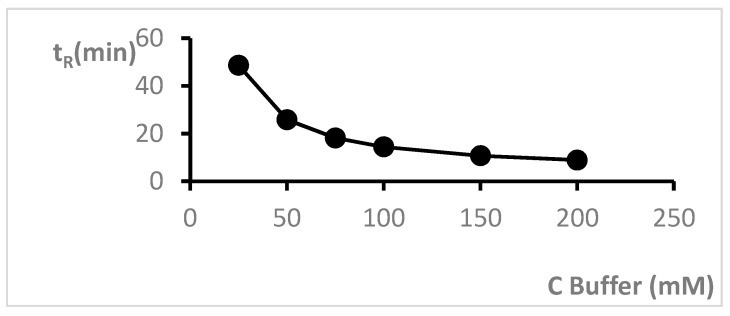
Dependence of t_R_ vs. formate buffer concentration obtained on Luna 5 μm, SCX 100A, 150 × 4.6 mm column with the mobile phase containing ACN and formate buffer at pH 4.0.

In the next step of experiments, dependence of retention, peaks’ shape and systems’ efficiency in relation to the concentration of formate buffer were examined. The investigation was performed in a range of concentrations from 25 to 200 mM. The increase in concentration of the buffer caused a significantly decrease in retention (Figure 5). In the system containing 25 mM of the buffer, cytisine was eluted after 48.62 min, whereas in system with addition of 200 mM of the buffer, cytisine was eluted after 8.91 min. With increasing buffer concentration, the N/m values were reduced from 75,500 (in the system with the lowest concentration of buffer) to 51,200 (in the system with the highest concentration) (Table 2). The symmetry of peaks obtained for cytisine changed only very slightly when the buffer concentration changed, and As was 0.97 in most examined systems. The results show that application of the SCX column and mobile phases containing acetonitrile and formate buffer is suitable for analysis of cytisine.

Cytisine is a polar basic compound that is easily dissociated in acidic solutions and for this reason can be easily retained in an ion-exchange stationary phase. The application of ion exchange chromatography enables the strongest retention to be obtained among all tested chromatographic systems, which is especially important in the analysis of biological samples because it allows good separation of cytisine from the components of the matrices.

Based on the results obtained considering retention, peaks’ symmetry and systems’ efficiency, determination of cytisine in biological samples and pharmaceutical formulation with diode array and fluorescence detection was performed on the SCX column with the mobile phase containing 15% of ACN and 100 mM of formate buffer at pH 4.0.

Application of the Polar-RP stationary phase allows results to be attained in a short time, using a mobile phase containing a small amount of organic solvent or only water without any additions. The above property makes the systems with the Polar-RP stationary phase a good alternative for application in green chromatography. Moreover, for LC-MS analysis it is possible to use the Polar-RP phase with smaller dimensions, which leads to reduced solvent consumption (Synergi HST Polar RP, Table 1).

### 2.2. Optimisation of Sample Pre-Treatment Method

**Figure 6:**
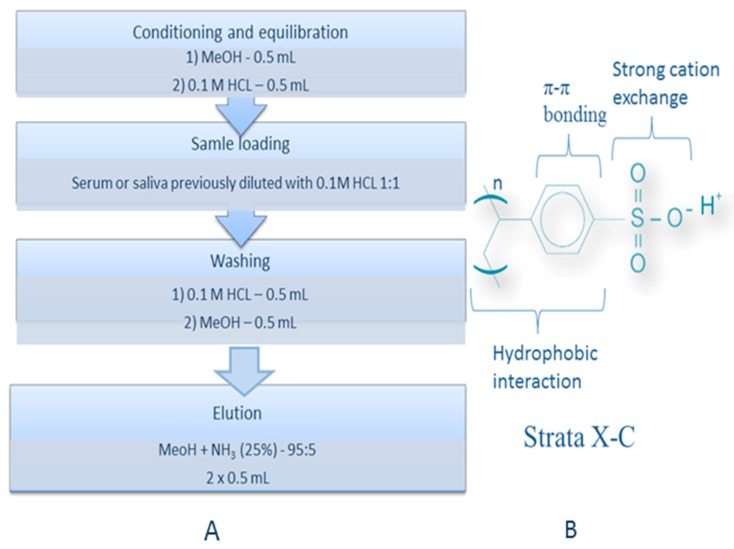
The scheme of the SPE procedure applied for the preparation of human serum and saliva samples (**A**) and scheme of Strata X-C phase with description of retention mechanisms (**B**).

Different sample pre-treatment methods were tested: protein precipitation (with acetone, MeOH or ACN) and solid phase extraction (SPE) using cartridges with C18, HLB or strong cation exchange sorbents (Strata X-C). Protein precipitation did not get rid of interference. Reverse phase retention characteristic for C18 or HLB is not suitable for cytisine due to its polar character leading to small recoveries (below 70% in all tested systems). Besides, protein precipitation and SPE using C18 or HLB columns led to poor purification of samples from interfering substances, which was particularly noticeable in MS detection. For these reasons, application of the above extraction methods was not suitable for LC-MS analysis using selected chromatographic systems. Satisfactory results were obtained by the use of columns containing ion exchange sorbent. Strata-X-C is a polymeric strong cation exchange sorbent that couples a hydrophobic skeleton with a sulfonic acid functional group. Strata-X-C columns proved to be the most suitable for the preparation of serum and saliva samples. Using these SPE columns, good recoveries for cytisine (over 90%) were obtained. For this reason, SPE procedure on SCX columns was applied for preparation of serum and saliva samples for further investigations. Procedure of SPE is presented in Figure 6A. The analytes were retained by several different mechanism: strong cation exchange, π–π bonding and hydrophobic interaction (Figure 6B). The obtained absolute recovery for samples spiked by 100 ng mL^−1^ of cytisine was 93.50% ± 8.29% (*n* = 3) for serum and 97.85% ± 3.42% (*n* = 3) for saliva samples.

**Figure 7:**
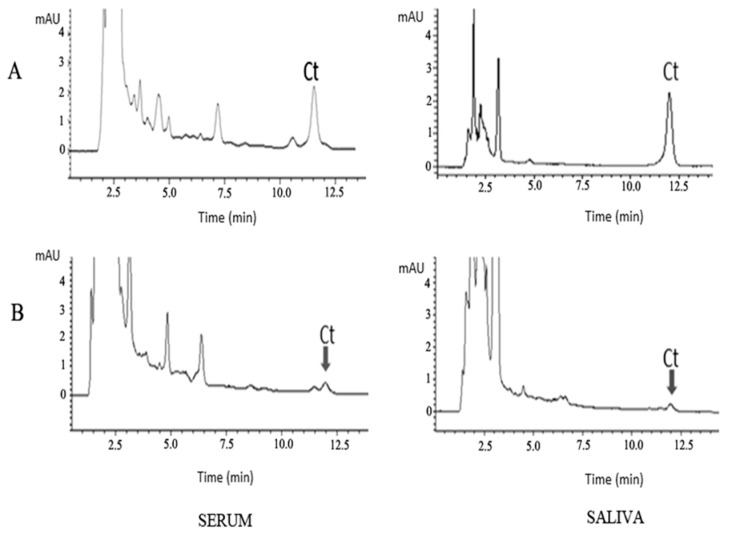
Chromatograms obtained for cytisine (Ct) on Luna 5 μm, SCX 100A, 150 × 4.6 mm column with mobile phase containing 15% ACN and 100 mM of formate buffer at pH 4.0 in serum and saliva samples: (**A**) spiked with 100 ng/mL of cytisine, (**B**) samples from patients treated with drug. Samples were collected 2 h after single oral dose administration of 4.5 mg (saliva) or 1.5 mg (serum).

Depending on the detection method used, different sample volumes were applied to SPE procedure. One millilitre of serum or saliva was used for HPLC-DAD analysis. The samples were concentrated to 50 μL. Examples of obtained chromatograms are presented in Figure 7. Lower serum or saliva volume, 250 µL, was used for LC-MS/MS analysis. After SPE procedure, samples were also concentrated to 50 µL. The obtained absolute recovery for samples spiked by 100 ng mL^−1^ of cytisine was 97.85 % (±3.42%), *n* = 3 for saliva samples and 93.50 % (±8.29%), *n* = 3 for serum samples. These obtained recoveries are higher that presented in literature for biological samples in humans [22,23,24,25,26,30,31].

### 2.3. Analysis of Cytisine in Serum and Saliva Samples

#### 2.3.1. Analysis of Cytisine in Serum and Saliva Samples by HPLC-DAD

The performed optimisation enables the selection of chromatographic conditions for analysis of cytisine in serum and saliva samples spiked with 100 ng/mL of cytisine, and samples from patients treated with the drug. Taking into account the results in terms of separation selectivity of analytes, selectivity of separation in relation to the matrix components, peaks’ symmetry and systems’ efficiency, the SCX column with the mobile phase containing 15% acetonitrile and formic buffer at pH 4.0 was selected for determination of cytisine in biological samples. The sensitivity of HPLC-DAD and HPLC-FLD methods is similar, requires the use of larger sample volumes and is suitable for higher concentrations of cytisine. The LOD for the HPLC-DAD method (calculated as above) was 5.99 and 6.20 ng/mL for saliva and serum, respectively. Detection of cytisine in saliva by HPLC-DAD was possible 2 h after dose (maximum concentration of cytisine in serum [31]) of 4.5 mg. Detection of the drug in saliva was not possible after single dose of 1.5 mg of cytisine; however, it was carried out for serum samples after the same dose. In both cases, the resulting signals were rather low. Better results can be obtained at higher concentrations of cytisine after multiple doses of the drug. Examples of obtained chromatograms for cytisine in serum and saliva samples are shown in Figure 7.

#### 2.3.2. Analysis of Cytisine in Serum and Saliva Samples by HPLC-MS

**Figure 8:**
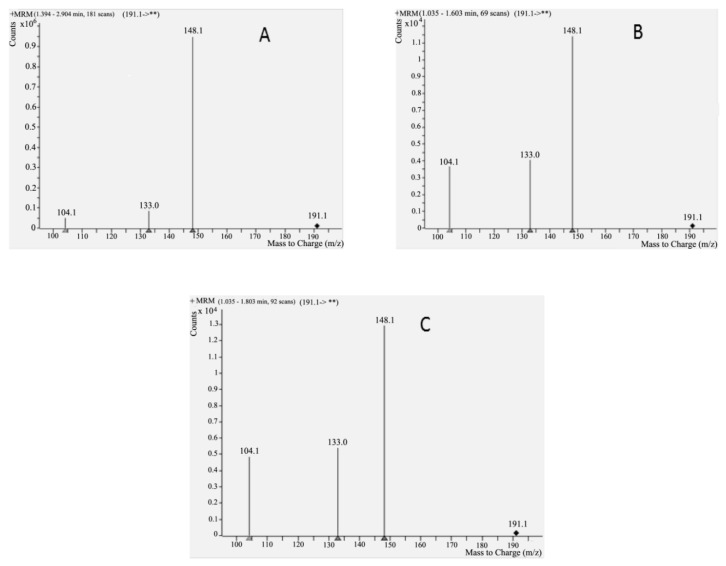
MS spectra obtained for: Cytisine standard (**A**), cytisine in saliva sample collected 1 h after single oral dose administration of 1.5 mg (**B**) and cytisine in saliva sample collected 24 h after single oral dose administration of 1.5 mg (**C**). Stationary phase: Synergi 2.5 µL Polar-RP 100 Å; mobile phase – methanol: water + 0.1% HCOOH (82:18); flow: 0.3 mL/min; volume injected: 10 µL.

Cytisine in saliva samples was detected for the first time (LC-QqQ-MS: multiple reaction monitoring (MRM) mode parameters for the cytisine are presented in Table 3 and appropriate MS spectrum is presented in Figure 8). The detection of cytisine in saliva by MS was possible even during the 24 h after single dose of 1.5 mg. Limit of detection (LOD) was 0.29 and 0.30 ng/mL for saliva and serum, respectively. LOD was calculated as signal:noise ratio 3:1. LC-MS/MS is the most suitable for cytisine analysis in biological samples due to sensitivity of the assays.

Selectivity was evaluated by analysing the serum and saliva samples from different sources (including smoking and non-smoking participants) to investigate the potential interferences with the signals of drug. No interference was found at the specific retention time of analyte in spiked samples in comparison to blank serum/saliva. Cytisine peak was fully separated from components of the matrix.

### 2.4. Analysis of Cytisine in Pharmaceutical Formulation

The HPLC-DAD method was applied for determination of cytisine in capsules (Desmoxan^®^). Formulation was stored under conditions recommended by the manufacturer. Different columns were tested for cytisine analysis in pharmaceutical formulation. The highest retention was obtained on the SCX stationary phase, which allowed for retention modification in a wide range. It seems to be suitable for application in the quantification of cytisine in pharmaceutical formulations and for potential forced degradation and stability indicating studies. To date, as reported in literature [23], determination of cytisine in formulations was performed using C18 stationary phase, which gives poor retention due to polar character of cytisine molecule. Interestingly, the stationary phase for analysis of cytisine in formulations was also Polar-RP, which allowed results to be obtained in a short time (less than 6 min) using the mobile phase containing a small amount of organic solvent or only water without any additions. The above property makes the systems with Polar-RP stationary phase a good alternative for application in green chromatography due to the use of mobile phases with very small amounts of organic modifiers or without the addition of organic modifiers. For example, the HPLC-DAD analysis of cytisine obtained from capsules were performed using systems containing only water with (or without) addition of formic acid or water and a small amount of organic modifier (methanol or acetonitrile) with formic acid. The quantifications were performed using the SCX column and the mobile phase containing acetonitrile (15% *v*/*v*) and ammonium formate buffer (85% *v*/*v*, 100 mM, pH 4.0). The calibration curve equation was as follows: y = 49,881 (±148) x − 5654 (±387). The linearity range of cytisine for 0.1–50 μg/mL was obtained with LOD and limit of quantification (LOQ) 0.5 and 1.52 μg/mL, respectively. Linearity was verified for this method by using values for coefficients of determinations (R^2^) obtained from linear standard curves. The R^2^ of the calibration curve was 0.9999. Obtained chromatographic parameters (for target cytisine concentration of 20 µg/mL) were as follows: t_R_ = 12, As = 0.96, N/m = 60,840. The result of the assays undertaken yielded 96.27% ± 0.31% of label claim for cytisine. The concentration of the drug was 19.25 ± 0.06 µg/mL, which corresponds to 1.44 mg per capsule. Any chromatographic interference from the capsule excipients was detected. Peak purity index was confirmed by comparison of analysed investigated cytisine UV spectra with the spectra of drug standard. Peek purity index for analysed cytisine from formulation was close to 1.0 in all the studied cases. A peak purity index of 1.0 indicates that the compared spectra are identical.

## 3. Materials and Methods

### 3.1. Chemicals and Reagents

Standard of cytisine and Desmoxan^®^ (pharmaceutical formulation, micronized capsules 1.5 mg) were obtained from Aflofarm (Pabianice, Poland). Methanol (MeOH), acetonitrile (ACN) of chromatographic quality, diethylamine (DEA), acetic acid (99–100%), formic acid (98–100%), sodium acetate, ammonium acetate, ammonium formate and water for LC-MS were purchased from Merck (Darmstadt, Germany). Water for HPLC-DAD and HPLC-FID analysis was double distilled. Sodium methanesulfonate, sodium hexafluorophosphate and sodium chlorate were obtained from Sigma-Aldrich (Schnelldorf, Germany). Sodium dihydrogen phosphate and sodium chloride were from Polish Reagents (POCh) (Gliwice, Poland). 1-butyl-3-methylimidazolium tetrafluoroborate, sodium tetrafluoroborate were of analytical grade obtained from Merck (Darmstadt, Germany); 1-octyl-3-methylimidazolium tetrafluoroborate, 1-ethyl-3-methylimidazolium tetrafluoroborate, 1-hexyl-3-methylimidazolium tetrafluoroborate, 1-butyl-3-methylimidazolium nitrate, 1-dodecyl-3-methylimidazolium tetrafluoroborate, 1-butyl-3-methylimidazolium methanesulfonate, 1-butyl-3-methylimidazolium chlorate, 1-butyl-3-methylimidazolium dihydrogen phosphate, and 1-butyl-3-methylimidazolium hexafluorophosphate were obtained from Hangzhou Sage Chemical (Hangzhou, China).

### 3.2. Apparatus and HPLC Conditions

The chromatographic analysis using different techniques: HPLC-DAD, HPLC-FID or HPLC-MS/MS was performed on columns presented in Table 1. HPLC conditions are described below. The efficiency of chromatographic systems was calculated as theoretical plate number expressed as N/m (per meter of column) and peak symmetry as asymmetry factor (As).

#### 3.2.1. HPLC-DAD Conditions

Analysis was performed using an LC-10ATVP Shimadzu liquid chromatograph (Shimadzu Corporation, Canby, OR, USA) equipped with a Shimadzu SPD-M20A detector (Shimadzu Corporation, Canby, OR, USA). Flow rate was 1 mL/min. Detection was carried out at a wavelength of 303 nm. All chromatographic measurements were controlled by a CTO-10ASVP thermostat (Shimadzu Corporation, Canby, OR, USA). Extracts were injected into the columns using the Rheodyne 20 μL injector. The DAD detector was set in the 200–800 nm range. Data acquisition and processing were carried out with a LabSolutions software (Shimadzu Corporation, Kyoto, Japan).

#### 3.2.2. HPLC-FLD Conditions

LC analysis was performed using an Agilent Technologies 1200 HPLC system (Agilent Technologies, Waldbronn, Germany) with a quaternary pump (Agilent Technologies, Tokyo, Japan) and 1260 FLD Spectra fluorescence detector (Agilent Technologies, Waldbronn, Germany). The samples were injected onto chromatographic column using a Rheodyne 20 μL manual injector. The column was set at thermostat temperature of 22 °C. The mobile phases were delivered at a constant 1 mL/min flow. FLD detection was performed at an excitation wavelength of 300 nm.

#### 3.2.3. HPLC-MS/MS Conditions

Chromatographic analysis was performed using HPLC (Agilent 1260; Agilent Technologies, Boblingen, Germany). The mass spectral analysis was performed on a 6460 triple quadrupole mass spectrometer from Agilent Technologies (Santa Clara, CA, USA) equipped with an Jet Stream ESI interface operating in positive ion mode, with the following set of operation parameters: capillary voltage, 3500 V; nebulizer pressure, 45 psi; drying gas flow, 5 L/min; drying gas temperature, 300 °C; sheath gas temperature, 250 °C, and sheath gas flow, 11 L/min. Quadrupole 1 was fixed at a set parent ion, quadrupole 2 was used as a collision chamber to induce fragmentation, and quadrupole 3 was fixed at a set daughter ion. Identification of cytisine was carried out on the basis of multiple reaction monitoring (MRM) (Table 3).

### 3.3. Serum and Saliva Samples Collection

Serum and saliva samples were collected from smoking and non-smoking patients with no liver and kidney abnormalities taking cytisine in single dose of 1.5 mg (or 4.5 mg for detection test in saliva by HPLC-DAD) or using the dosing regimen of one capsule six times per day through three days). The collection of samples was performed in various periods of time after taking a dose of the drug by the patient.

Participants did not brush their teeth for 2 h before the test and did not eat at last 1 h or did not drink for 20 min before sample collection. Participants did not drink caffeinated beverages (coffee, energy drinks) for 12 h before the test. Ten minutes before giving saliva, the participants rinsed the mouth three times with a small amount of deionized water. The saliva was collected in a sterile plastic container and then frozen at −80° C. The collected blood was incubated at room temperature (15–24 °C) for 30 to 40 min. Then, blood was centrifuged for 15 min at 2000 rpm. The obtained serum was transferred to a sterile plastic tube with a sealed stopper. The serum sample was frozen at −80 °C.

The study protocol was approved by the Bioethical Committee of the Medical University of Lublin (approval number KE-0254/165/2018).

### 3.4. Extraction Procedure for Isolation of Investigated Drugs from sErum and Saliva

Protein precipitation was performed using ACN, MeOH or Acetone in different amounts. Solid phase extraction was performed using BAKERBOND^TM^ spe Octadecyl (C18) J.T. Baker cartridges, Oasis HLB cartridges or Strata X-C cartridges (30 mg/mL, Phenomenex) and SPE chamber—Baker SPE—12G (J.T. Baker, Philipsburg, USA). For final analysis, the SPE method with Strata X-C cartridges was developed. The scheme of the SPE procedure is presented in Figure 6.

The extraction recovery at each concentration was calculated by use of the following equation:

% Recovery = Peak area (extraction)/Peak area (standard injection) × 100(1)

### 3.5. Preparation of Stock Solution and Working Solutions

The stock standard solution of cytisine was prepared in methanol at a concentration of 0.2 mg/mL by dissolving 10 mg of the drug in 50 mL of methanol, and stored at −20 °C, protected from light. The working standard solutions of cytisine were prepared by diluting of the above mentioned stock solution in methanol before analysis.

### 3.6. Procedure for Analysis of Cytisine in Pharmaceutical Formulation

Ten capsules were weighted, and the average capsule mass was calculated. A quantity of powder equivalent to one capsule containing 1.5 mg of cytisine was transferred into a 50 mL volumetric flask. To this flask, 50 mL of methanol were added, and the solution was shaken for 10 min. From this solution, aliquots of appropriate volume were transferred to 10 mL volumetric flasks and diluted with methanol to obtain a final concentration of 20 µg/mL of cytisine. Twenty microliters of solution were injected into the column. The measurements were performed in triplicate.

Standard curve was prepared using seven concentration: 0.1, 0.5, 1, 10, 20, 30, 40 and 50 μg/mL in triplicate LOD and LOQ were calculated according to the formula: LOD = 3.3 (SD/S), and LOQ = 10 (SD/S), where SD is the standard deviation of response (peak area) and S is the slope of the calibration curve.

Linearity was determined by injections of above solutions in triplicate. The average peak areas were plotted against concentrations. The linearity was evaluated by calculating coefficient of correlation, slope and intercept.

The specificity was evaluated to ensure that there was no interference from the excipients present in the capsules. Peak purity was confirmed by comparison of investigated drug UV spectra (Shimadzu SPD-M20A detector (Shimadzu Corporation, Canby, OR, USA)) with the spectra of standard.

## 4. Conclusions

For the first time, chromatographic systems for analysis of cytisine were optimised with the application of a large number of various stationary phases and mobile phases of different compositions. Cytisine, as a very polar compound, is very poorly retained in usually used RP systems, and especially weak retention was observed on most often used alkyl-bonded stationary phases. The application of columns with phenyl phases generates π–π interaction and resulted in the increase of retention in most applied mobile phases. A significant increase in cytisine retention and improvement of peak shape was obtained on the Polar RP column.

HILIC systems that have not been used previously can also be useful for determination of cytisine. The best results in terms of retention, symmetry of peaks and system efficiency was obtained on the HILIC-A column with the mobile phase containing 80% ACN and formate buffer at pH 4.0.

For the first time, for analysis of cytisine, ion-exchange chromatography was successfully applied. The strongest retention, excellent shape of peaks and high system efficiency were obtained for cytisine using the IEC method on the SCX column with mobile phases containing ACN and phosphoric or formate buffers.

Based on our experiments, it can be concluded that the most suitable system for analysis of cytisine by HPLC-DAD and HPL-FLD is the system containing the SCX column and the mixture of ACN and formate buffer at pH 4.0. The symmetrical peaks and high N/m values were also obtained on the Polar RP column with mobile phases containing addition of DEA and salts. The application of systems with high concentration of buffer, salt or DEA was less advantageous for the analysis of cytisine by HPLC-MS; therefore, the best sensitivity by this method was obtained when the system containing the Polar RP column and the mixture of MeOH, water and formic acid as eluent were used.

The optimisation of chromatographic systems enabled the selection of the most optimal conditions for cytisine analysis in samples of human serum, saliva and pharmaceutical formulation. Due to the different separation principles, RP on phenyl stationary phases, IEC and HILIC can be successfully applied for cytisine analysis as an alternative to the most commonly used RP methods carried out on alkyl-bonded stationary phases. Detection of cytisine can be successfully performed using DAD, FLD or MS systems depending on the required sensitivity of assays.

Application of the SPE method with Strata X-C cartridges containing a strong cation exchange sorbent that couples a hydrophobic skeleton with a sulfonic acid functional group for sample preparation, and optimal HPLC systems for analysis, allowed good recoveries to be obtained for cytisine isolated from biological samples.

Detection of cytisine in human serum was performed for the first time, analysis of the alkaloid in serum and pharmaceutical formulation by IEC also was performed for the first time.

The research carried out are the first such extensive chromatographic studies on cytisine. The obtained results can be fundamental for the development of analytical methods for quantitative and qualitative analysis of cytisine in various type of samples by liquid chromatography coupled with modern detection techniques. The findings can be widely used in pharmaceutical, toxicological and environmental analysis due to the constantly growing role of cytisine and its derivatives in various areas of medicine and science.

## Figures and Tables

**Table 1:** List of tested columns and their physicochemical properties.

Column	Functional Group	Length (mm)	(Inner Diameter) I.D. (mm)	Endcapped	Particle Size (μm)	Pore Size (Å)	Surface Area (m^2^/g)	Carbon Load (%)	Recommended pH Range
**Synergi Polar RP**	Ether-linked phenyl	150	4.6	Proprietary (polar group)	4	80	475	11	1.5–7.0
**Synergi HST Polar RP**	Ether-linked phenyl	100	2	Proprietary (polar group)	2.5	100	475	11	1.5–7.0
**CSH Phenyl-Hexyl**	Phenyl-Hexyl	150	4.6	Proprietary	5	130	185	15	1.0–11.0
**Synergi Hydro-RP**	Octadecyl (C18)	150	4.6	Proprietary (polar group)	4	80	475	19	1.5–7.5
**ACE HILIC-A**	Proprietary SIL	150	4.6	NO	5	100	300	-	2.0–7.0
**ACE HILIC-B**	Proprietary Aminopropyl	150	4.6	NO	5	100	300	4	2.0–7.0
**ACE HILIC-C**	Proprietary Polyhydroxy	150	4.6	NO	5	100	300	7	2.0–7.0
**Luna SCX**	Benzene Sulfonic Acid	150	4.6	NO	5	100	400	0.55 Sulphur Load	2.0–7.0

**Table 2:** Retention time (t_R_), asymmetry factor (As) and theoretical plate number per meter (N/m) values obtained for cytisine at concentration 10 µg mL^−1^ on various columns with different mobile phases; * fuzzy peak.

Column	Mobile Phase	t_R_	As	N/m
Hydro RP	5% MeCN + H_2_O + 0.1% HCOOH	1.76	*	*
	5% MeOH + H_2_O + 0.1% HCOOH	1.85	*	*
	H_2_O + 0.1% HCOOH	1.92	*	*
	5% MeOH + 20% acetate buffer at pH 3.5 H_2_O + 0.025ML^−1^ DEA	1.91	*	*
	20% acetate buffer at pH 3.5H_2_O + 0.025ML^−1^ DEA	1.56	*	*
	5% MeCN + H_2_O + 0.025ML^−1^ NaBF_4_	2.98	*	*
	10% MeCN + H_2_O + 0.025ML^−1^ ILBF_4_	2.96	*	11,120
	H_2_O + 0.025ML^−1^ IL BF_4_	3.46	*	12,740
Phenyl-Hexyl	5% MeCN + H_2_O + 0.1% HCOOH	1.23	*	*
	5% MeOH + H_2_O + 0.1% HCOOH	1.38	*	*
	H_2_O + 0.1% HCOOH	1.55	*	*
	5% MeCN + 20% acetate buffer at pH 3.5 H_2_O + 0.025ML^−1^ DEA	3.49	0.73	13,200
	5% MeOH + 20% acetate buffer at pH 3.5 H_2_O + 0.025ML^−1^ DEA	3.64	0.60	*
	20% acetate buffer at pH 3.5H_2_O + 0.025ML^−1^ DEA	5.29	0.64	10,070
	H_2_O + 0.025ML^−1^ NaCl	*		
	H_2_O + 0.025ML^−1^ IL Cl	2.45	1.04	12,140
	H_2_O + 0.025ML^−1^ NaBF_4_	6.69	1.60	13,390
	H_2_O + 0.025ML^−1^ IL BF_4_	3.25	1.08	12,240
Polar RP	5% MeCN + H_2_O + 0.1% HCOOH	3.30	*	*
	5% MeOH + H_2_O + 0.1% HCOOH	4.10	*	*
	H_2_O + 0.1% HCOOH	5.31	1.50	37,260
	5% MeCN + 20% acetate buffer at pH 3.5 H_2_O + 0.025ML^−1^ DEA	3.49	1.31	31,830
	5% MeOH + 20% acetate buffer at pH 3.5 H_2_O + 0.025ML^−1^ DEA	4.49	1.33	23,750
	20% acetate buffer at pH 3.5 + H_2_O + 0.025ML^−1^ DEA	5.90	1.47	21,030
	5% MeCN + H_2_O + 0.025ML^−1^ NaH_2_PO_4_	3.76	1.22	41,870
	H_2_O + 0.025ML^−1^ IL H_2_PO_4_	2.66	1.97	16,610
	5% MeCN + H_2_O + 0.025ML^−1^ NaMeSO_3_	4.07	1.20	44,320
	H_2_O + 0.025ML^−1^ IL MeSO_3_	2.10	1.93	13,920
	5% MeCN + H_2_O + 0.025ML^−1^ NaCl	3.91	1.19	44,570
	H_2_O + 0.025ML^−1^ IL Cl	5.91	0.81	22,400
	5% MeCN + H_2_O + 0.025ML^−1^ NaBF_4_	5.19	1.11	55,120
	H_2_O + 0.025ML^−1^ IL BF_4_	3.59	0.66	7250
	5% MeCN + H_2_O + 0.025ML^−1^ NaClO_4_	6.33	1.16	56,230
	H_2_O + 0.025ML^−1^ IL ClO_4_	5.58	1.15	45,580
	5% MeCN + H_2_O + 0.025ML^−1^ NaPF_6_	8.95	1.04	69,470
	H_2_O + 0.025ML^−1^ IL PF_6_	3.56	1.02	28,850
HILIC A	80% MeCN + H_2_O + 0.1 ML^−1^ HCOONH_4_	3.91	*	*
	80% MeCN + formate buffer at pH 4.0	6.82	1.08	56,940
HILIC B	80% MeCN + H_2_O + 0.1 ML^−1^ HCOONH_4_	2.96	0.80	10,330
	80% MeCN + formate buffer at pH 4.0	9.61	1.51	4680
HILIC N	80% MeCN + H_2_O + 0.1 ML^−1^ HCOONH_4_	4.38	1.25	33,290
	80% MeCN + formate buffer at pH 4.0	4.74	1.11	40,890
SCX	15% MeCN + phosphoric buffer at pH 2.5	5.13	0.96	48,670
	15% MeCN + 25 mM formate buffer at pH 4.0	48.62	1.04	75,520
	15% MeCN + 50 mM formate buffer at pH 4.0	25.82	0.97	66,360
	15% MeCN + 75 mM formate buffer at pH 4.0	18.16	0.97	61,720
	15% MeCN + 100 mM formate buffer at pH 4.0	14.44	0.97	58,780
	15% MeCN + 150 mM formate buffer at pH 4.0	10.74	0.97	53,810
	15% MeCN + 200 mM formate buffer at pH 4.0	8.91	0.98	51,200

**Table 3:** LC-QqQ MRM transition parameters for the cytisine.

Precursor Ion	Fragmentor	Product Ion	Collision Energy
191.12	148	148.1	20
		133.0	36
		104.1	60
